# Assessing active thumb palmar and radial abduction in persons with thumb carpometacarpal osteoarthritis via intermetacarpal distance methods: an exploration of validity, reliability, and precision

**DOI:** 10.1007/s10067-026-08003-3

**Published:** 2026-02-25

**Authors:** Halil Ibrahim Ergen, Karl Dischinger, Corey W. McGee

**Affiliations:** 1 Department of Physical Therapy and Rehabilitation, Faculty of Health Sciences, Gaziantep University, 27050 Gaziantep, Türkiye; 2 Program in Occupational Therapy, College of Pharmacy, University of Minnesota, Minneapolis, MN USA; 3 Program in Rehabilitation Science, Medical School, University of Minnesota, Minneapolis, MN USA

**Keywords:** Carpometacarpal joints, Intermetacarpal distance, Osteoarthritis, Reliability, Reproducibility of results, Thumb

## Abstract

**Background:**

Thumb carpometacarpal osteoarthritis (CMC1 OA) is a prevalent and disabling rheumatological condition. One impairment commonly described in the context of CMC1 OA is a loss of radial (RABD) and palmar (PABD) thumb abduction. Traditional goniometric measures are unreliable and poorly correlated with functional limitations. The intermetacarpal distance (IMD) method, using digital calipers, shows promise for better reliability, but its validity and the feasibility of using tape measures remain untested.

**Objective:**

To evaluate the construct validity, test–retest reliability, and precision of IMD-based thumb abduction measurements using calipers and tape in individuals with non-operative, radiographically confirmed CMC1 OA.

**Methods:**

Forty participants underwent standardized IMD assessments using both caliper and tape methods across two sessions, 2 weeks apart. Three trials were recorded per session. Reliability (ICC_2,3_), precision (SEM, MDC, MDC%), and construct validity (correlation with Michigan Hand Questionnaire [MHQ]) were analyzed.

**Results:**

Reliability (ICC_2,3_) ranged from 0.90 (PABD-tape, 1 trial) to 0.97 (RABD-caliper, 2–3 trials). All methods had acceptable precision (MDC% < 22); PABD-caliper (3 trials) showed excellent precision (MDC% = 9.1). RABD-caliper and RABD-tape showed strong, significant correlations with MHQ ADL scores (*r* = .38, *p* < .05; *r* = .40, *p* < .01). PABD methods showed weak or non-significant associations.

**Conclusion:**

Caliper and tape-based IMD measurements offer similar reliability, but averaging multiple trials improves precision. RABD-IMD methods demonstrate moderate construct validity, supporting their use in clinical assessment of CMC1 OA. Standardized, repeatable IMD assessments may enhance monitoring and care planning in thumb CMC1 OA.

**Table:** 

**Key Points** In persons with carpometacarpal osteoarthritis: • *Caliper and tape intermetacarpal distance measures of thumb radial and palmar abduction demonstrate excellent test-rest reliability and acceptable precision.* • *Averaging two or three intermetacarpal distance measurements is recommended over a single measure due to superior precision.* • *Radial abduction (via the intermetacarpal distance) is strongly associated with self-reported disability and may be more clinically meaningful than palmar abduction.*

## Introduction

Thumb carpometacarpal joint (CMC1) osteoarthritis (OA) is the second most common and the most symptomatic form of hand OA [1]. Of the different risk factors, the most well-known are age and female sex. Above the age of 50 years, the prevalence of radiographic OA was 5.8% for men and 7.3% for women, whereas above the age of 80 years, these rates were 33.1% and 39.0% for men and women, respectively [1]. In addition to the age and gender factors shown in this study, several risk factors for the development of CMC1 OA have been reported, such as repetitive use of the thumb and being postmenopausal [1]. Recent studies report that the prevalence of isolated radiographic CMC1 joint arthritis can reach approximately 20% in older women [2].

The thumb can move in different directions during activities of daily living through concerted movements of the CMC1, metacarpophalangeal (MCP1) and interphalangeal (IP) joints [3]. CMC1 OA is one of the most common clinical conditions that limit the movement of the thumb [4] and is characterized by a combination of decreased cartilage thickness, reduced range of motion (ROM), increased ligament laxity, and subluxation [5, 6]. In addition, the proprioception of these individuals is impaired, and it has been reported that these deficits may lead to problems in performing some tasks that may be important for activities of daily living [7, 8].

Adduction contracture, which is one of the frequently seen symptoms in these individuals, restricts the movements of the thumb, decreasing the distance between the thumb and the tip of the little finger. This reduces the range of motion of the CMC1 joint and results in a decreased thumb web space [9]. Adduction contracture progresses over time, along with other commonly reported symptoms such as thumb pain, loss of pinch strength, decreased thumb motion, and functional limitations [10]. As such, restoring the thumb webspace is a common treatment consideration in the context of CMC1 OA [11].

Thumb motion is difficult to measure due to its complex three-joint dynamic mobility and complex tasks [12]. Researchers have used various methods with varying reliability to measure change in web space. Angular (e.g., Pollexograph-thumb, Pollexograph-metacarpal, and intermetacarpal methods) and distance-based methods (e.g., intermetacarpal distance and interphalangeal distance) as well as technological methods (e.g., 3D camera systems and wearable gyroscope) are available to measure thumb abduction [9, 13–15]. These methods have some disadvantages related to their clinical use [13]. One of these methods, and one of the most well-known, goniometry, traditionally used to assess ROM at various sites throughout the body, has also been used to assess thumb ROM [13, 16]. However, the reliability of goniometry in the assessment of thumb palmar abduction (PABD) in healthy and affected hand samples was reported to be poor [16, 17], whereas it was reported to be moderate in the measurement of radial abduction (RABD) in individuals with CMC1 OA [17]. The reliability intervals of the measurement methods used have varied widely [13, 14], and no consensus has been reached on which method is the optimal [13, 14, 18]. As a result of our research using the method of McGee et al. [9] who first described the use of the intermetacarpal distance (IMD) method in the measurement of thumb PABD and RABD in individuals with CMC1 OA, we concluded that the measurement of thumb RABD with the IMD method had acceptable agreement, excellent test–retest reliability, and acceptable-to-excellent precision [19]. There is no research on the test–retest reliability of IMD measurements of PABD in individuals with CMC1 OA. It is critical to determine the test–retest reliability, which is one of the important parameters in the evaluation of patient prognosis and treatment efficacy [20]. While the IMD is often assessed, with calipers [19], others have recently described the use of tape measurements in healthy adults [14]. Considering the ease of accessing tape measures and the excellent reliability in healthy adults described by Holzbauer et al. [14], it may have potential in the context of CMC1 OA. However, there is no information on the reliability of the IMD tape measure method in persons with CMC1 OA.

MCP joints are often enlarged in CMC1 OA and can fluctuate in size with inflammation. As a result, placing the tape on the dorsal surfaces of the 1 st and 2nd MC heads for PABD distance measurements may not accurately reflect the span of the webspace and could introduce measurement error [21, 22]. For that reason, an alternative approach is needed when using tape to measure PABD. Additionally, while a single trial of range-of-motion measurements is common practice, recent evidence suggests that using of the mean of two or three trials is more reliable than a single measurement of RABD using the caliper IMD method [19], which is frequently used in the measurement of normal range of motion. It is important to investigate whether this is also the case with the IMD tape measure method. Finally, there are presently no studies looking at the validity of the IMD method as a measure of functional ability in persons with CMC1 OA.

As such, the purposes of this study were to (1) establish methods for using a tape measure for assessing PABD using the IMD method in persons with CMC1 OA; (2) establish and compare the test–retest reliability, precision, and construct validity of the tape measure and caliper IMD methods for assessing PABD and RABD in persons with CMC1 OA; and (3) establish and compare the test–retest reliability and precision when using 1 trial, the mean of 2 trials, and the mean of three trials of each of these IMD approaches to assessing abduction in persons with CM1 OA.

## Methods

### Design

This study consisted of a single-group, prospective cohort design in which IMD measures were tested in two sessions over 2 weeks to assess test–retest psychometrics. The Consensus-based Standards for the Selection of health Measurement Instruments (COSMIN) [23] and The Strengthening the Reporting of Observational Studies in Epidemiology (STROBE) [24] guidelines were followed in the study’s design and the reporting of methods and findings.

### Participants

#### Ethical approval

 This study obtained ethical approval from the institutional review board (STUDY00006741).

#### Recruitment

Participants were identified via purposive sampling of electronic medical records (EMRs). Eligibility criteria included: (1) age 18 or older, (2) radiographically confirmed CMC1 osteoarthritis (OA), and (3) an EMR flag indicating interest in research participation. Eligible individuals received study invitations through the EMR’s “MyChart” portal.

Records of those who expressed interest were screened for the following exclusion criteria: (1) carpal tunnel syndrome, (2) neurological disorders causing upper extremity paresis, paralysis, or sensorimotor impairment, (3) thumb tenosynovitis/tendonitis, (4) rheumatological diseases, and (5) history of CMC1 arthroplasty. These conditions, while sometimes comorbid with CMC1 OA, were excluded to reduce confounding from non-OA-related symptoms or functional variability.

Candidates who passed this initial screening were contacted by phone for further screening and, when suitable, scheduled for an in-person visit. Additional exclusions were applied following phone screening and during in-person sessions when individuals (1) could not speak English, (2) had received or planned treatment for thumb pain (e.g., occupational therapy or intra-articular injection) within 6 weeks, (3) had fixed thumb adduction contracture with no appreciable CMC1 motion, or (4) had visual or cognitive impairments interfering with instruction-following or test participation. All participants provided written informed consent before data collection.

#### Testing personnel

The two therapists who served as raters had different academic educational backgrounds and clinical experience. Rater 1 was a physiotherapist with a Master’s (MS) and Doctorate (PhD) degree in Occupational Therapy and 11 years of clinical experience, while Rater 2 was an occupational therapist with a Doctorate in Occupational Therapy (OTD) and less than 1 year of clinical experience. Rater 1 was an international postdoctoral researcher at the University of Minnesota during the period of data collection. After returning to his academic institution in Türkiye, he contributed substantially to data analysis, interpretation, and manuscript preparation. The decision to use raters of variable experience when assessing the IMD is supported in the literature [9].

### Data collection procedures

The senior investigator gave two 1-h trainings to the assessors. The assessors administered caliper measurements via the standard protocol described by McGee et al. [9], while they took tape measurements based on a hybridization of Holzbauer et al. [14] and McGee et al. [9]. We collected data from individuals with radiographically confirmed CMC1 OA who accepted the research invitation in a university laboratory. The assessors evaluated either the affected thumb or the dominant thumb (if bilaterally affected). A questionnaire in the “motor domain” tests of the National Institutes of Health's “Toolbox” determined dominance [25]. We used the NEIKO 01407 A caliper and Baseline FEI 12–1210 flexible tape measure for measurements. The NEIKO 01407 A measures up to 150 mm, with a resolution of 0.01 mm and an accuracy of 0.02 mm. And the FEI12-1210 measures up to 150 cm and has a resolution of 0.1 cm.

The assessors first demonstrated standardized procedures for the RABD and PABD tests to the participants [9]. After the participants performed a practice trial, the assessors conducted three trials with 10 s between each. The assessors recorded these values in an encrypted electronic database. In line with hand therapists’ practice patterns, we invited participants for a return test approximately 2 weeks after the initial assessments [26]. Upon reassessment, the assessors remained blinded to the participants’ initial test results.

In addition to IMD caliper and tape measure measurements of thumb RABD and PABD, we collected demographic, pain, and disability data at the beginning of the first session to characterize the sample. We used the Numeric Pain Scale to assess pain [27] and the Thumb Michigan Hand Questionnaire (MHQ) to determine disability [28]. The datasets generated and/or analyzed during the current study are available from the corresponding author on reasonable request.

#### Measurement of thumb radial abduction

Given that previous studies had already described the test–retest reliability and precision of the caliper method, we assessed only the IMD of RABD via tape measurements. We palpated the first and second metacarpal heads and placed crosshairs on the mid-dorsal portion of each.

**Fig. 1:**
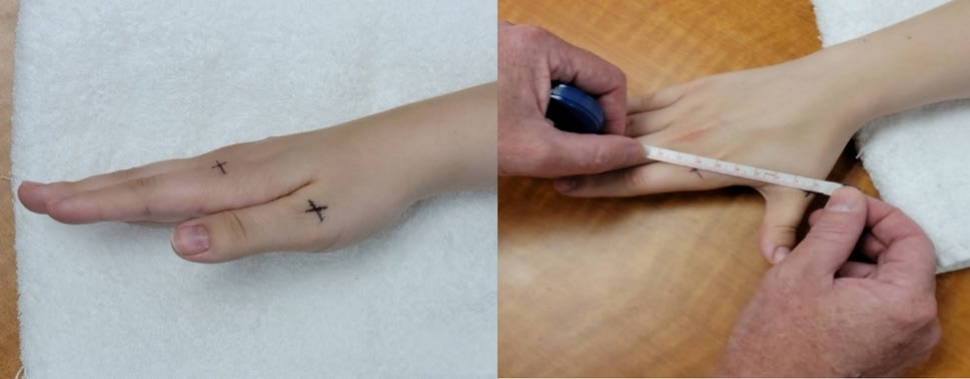
Dorsal mid-metacarpal markings of MC1 and MC2 and measurement of intermetacarpal distance during CMC1 radial abduction

We drew one line parallel to the metacarpophalangeal joint and another parallel to the shaft of the metacarpal. The bisection of these lines formed the cross marks. We then measured the distance (cm) between the center points of the cross marks on the dorsal heads of metacarpals 1 (MC1) and 2 (MC2) while asking the participant to perform maximum RABD. During the measurements, we supported the participant’s wrist and forearm with a towel to stabilize both without interfering with CMC1 motion (Fig. 1).

#### Measurement of thumb palmar abduction

**Fig. 2:**
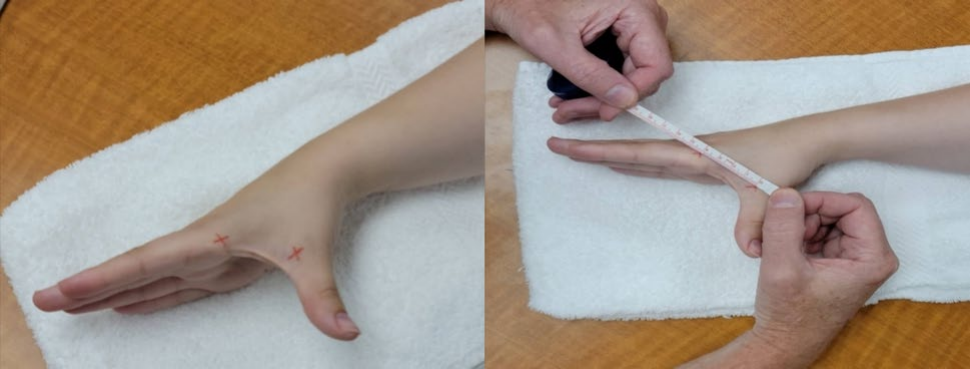
Mid-medial markings of MC1 and mid-lateral markings of MC2 and measurement of intermetacarpal distance during CMC1 PABD

We used the distance between the same points at MC1 and MC2 to measure PABD with the IMD-caliper method. During the measurement, we placed the forearm and wrist on the table in neutral positions, and the CMC1 motion occurred parallel to the surface of the table. For the measurement of PABD with the IMD-tape measure method, we followed the same testing procedures; however, instead of using dorsal landmarks, we placed crosshairs on the mid-medial surface of MC1 and the mid-lateral surface of MC2 to address the aforementioned concerns associated with dorsal-based tape measurements (Fig. 2).

### Disability

We measured disability using the Michigan Hand Outcomes Questionnaire (MHQ), a self-report tool specifically designed to assess upper extremity function, symptoms, pain, and disability across six domains: hand function, activities of daily living, work performance, satisfaction, aesthetics, and pain [29]. The MHQ generates scores ranging from 0 to 100, with higher scores on the first five domains indicating better function and outcomes and lower scores on the pain domain indicating less pain. The MHQ has demonstrated high reliability, validity, and responsiveness to change in adults with upper extremity musculoskeletal conditions, including arthritis [28, 29]. For this study, we reported only the disability score of the tested hand.

#### Pain severity

We assessed pain severity using a 0–10 Pain Numerical Rating Scale (NRS), where participants rated their pain intensity during daily activities over the past 24 h, with 0 indicating no pain and 10 representing the worst possible pain [30]. We chose the NPRS because it is a clinically relevant tool, widely validated for use in individuals with various musculoskeletal disorders, including CMC1 OA [30, 31].

### Sample size estimation

According to the sample size determination formula proposed by Walter et al. [32], 27 participants were needed for sufficient statistical power (expected reliability coefficient = 0.9, beta = 0.20; alpha = 0.05) [14] for reliability studies.

## Statistical analysis

Mean IMD values were calculated with data from initial and return assessments. Descriptive statistics were used to report IMD values and characterize the sample.

### Reliability and precision

The intraclass correlation coefficient (ICC_2,3_) was used to assess the test–retest reliability of the mean of one trial, the mean of two trials and the mean of three trials. ICC values between 0.4 and 0.75 were considered “good,” and those above 0.75 were considered “excellent” reliability [33]. Standard error of measurement (SEM) and minimum detectable change (MDC_95_) were used to determine the precision of one trial, the average of two trials, and the average of three trials.

The SEM reflects the measurement error of the instrument [34] and MDC_95_ is a tighter marker for precision and refers to the statistical estimate of the minimum clinically important difference [35]. These values were reported in millimeters for the IMD-caliper and centimeters for the IMD-tape measure. MDC values were normalized to express percentages of the range of IMD values and expressed as MDC%. The following formulas were used to perform these calculations:

- SEM = Standard Deviation of Mean * (√1-ICC)
- MDC_95_ = 1.96*√2 * SEM
- MDC% = (range of MDC/IMD values) *100

The MDC% value of less than 30 is considered “acceptable,” and less than 10 is considered “excellent” [36]. These statements about precision for IMD measurements in our study can be used by therapists to determine whether the change in a client’s measurements over time is due to an actual change in thumb abduction or an error and whether the detected change is clinically relevant [9].

#### Construct validity

A preliminary test of data normality, the Shapiro–Wilk test, was carried out to assess the normality of the distribution of IMD and MHQ data and inform the subsequent selection tests of bivariate associations. Based on these findings, the Pearson *R* coefficient would be used to determine associations if normally distributed and Spearman’s Rho would be used if non-normally distributed. Correlations that exceeded thresholds of 0.10, 0.20, and 0.30 would be interpreted as small, medium, and large effect sizes, respectively [37].

## Results

### Participants

A total of 150 patients who met the inclusion criteria were invited to participate in the study. Of the 69 individuals who responded to the invitation, 27 were excluded after review of medical records, and 40 of the 42 eligible participants completed the study. Attrition was due to one participant sustaining a distal radius fracture to the side assessed after the initial assessment and another being lost to follow-up. The data of all participants who completed the study (*n* = 40) were included in the analyses.

Table 1 shows the demographic data of the participants. Many of the participants were White (97.5%), non-Hispanic (97.5%), female (62.5%), and right-hand dominant (87.5%). The left hand was more commonly affected (42.5%), and the mean age of the participants was 65.9 (8.5) years with a mean Pain Numeric Rating Scale (NRS) score of 3.8 (2.4) and Total and Disability TDX mean scores were 33.0 (16.7) and 24.9 (17.3), respectively. The mean time between baseline and return sessions was 2 weeks (± 5.5 days).

**Table 1:** Sociodemographic and clinical characteristics of participants

Characteristics	*n*	*Counts (%)*	*Range*	*Mean (SD)*
**Sex**	40			
Male		15 (37.5)		
Female		25 (62.5)		
**Age** (years)	40		44–85	65.9 (8.5)
**Race**	40			
AI/AN		1 (2.5)		
White		39 (97.5)		
**Ethnicity**	40			
Hispanic		1 (2.5)		
Non-Hispanic		39 (97.5)		
**Affected hand**	40			
Right only		10 (25.0)		
Left only		17 (42.5)		
Bilateral		13 (32.5)		
**Dominance**	40			
Right		35 (87.5)		
Left		5 (12.5)		
**MHQ**	40			
Total		27.9 (17.7)		
Disability		71.5 (20.1)		
**Pain Severity (0–10 NRS)**	40		0–8	3.8 (2.4)
**Number of days between sessions**	40		7–34	13.9 (5.5)

*AI/AN* American Indian/Alaska Native, *n* number of participants, *NRS* Numerical Rating Scale, *MHQ* Michigan Hand Questionnaire

### Reliability and precision

Reliability and precision findings are presented in Table 2. The minimum and maximum reliability coefficients (ICC_2,3_) of the caliper and tape measure results for PABD and RABD were 0.90 (PABD tape measure-1 trial) to 0.97 (RABD caliper-2 and 3 trials), respectively. For RABD and PABD, the ICCs of the means of two and three trials for each measurement were the same and higher than that of the one trial. However, their 95% CIs contained the ICC values of one trial so the significance of these differences is questionable.

**Table 2:** Test–retest reliability and precision of the intermetacarpal distance method: descriptive data, intraclass correlation coefficients (ICC), standard error of measurement (SEM), and minimal detectable change (MDC_95_)

Measure	Trials	Time 1 M (SD) (Range)	Time 2 M (SD) (Range)	ICC (95%CI)	SEM (SEM%)	MDC^95^ (MDC%)
RABD-caliper	Mean 3 trials	65.77 (7.62) (46.89–87.91)	66.02 (7.92) (48.02–87.53)	0.97 (0.94–0.98)	1.35 (3.28)	3.7 (9.1)
	Mean 2 trials	65.74 (7.64) (46.93–87.97)	65.94 (7.96) (48.04–87.55)	0.97 (0.94–0.98)	1.35 (3.29)	3.7 (9.1)
	1 trial	65.58 (7.67) (46.90–87.88)	65.88 (8.03) (47.54–87.70)	0.94 (0.89–0.97)	1.92 (4.69)	5.3 (13.0)
RABD-tape	Mean 3 trials	6.69 (0.78) (4.87–8.87)	6.76 (0.79) (4.80–8.60)	0.96 (0.93–0.98)	0.15 (3.68)	0.41 (10.20)
	Mean 2 trials	6.69 (0.78) (4.87–8.87)	6.76 (0.79) (4.80–8.60)	0.96 (0.93–0.98)	0.15 (3.68)	0.41 (10.19)
	1 trial	6.68 (0.78) (4.80–8.80)	6.74 (0.78) (4.80–8.50)	0.93 (0.87–0.96)	0.21 (5.21)	0.58 (14.43)
PABD-caliper	Mean 3 trials	66.54 (7.19) (48.36–85.53)	66.58 (7.09) (47.63–81.06)	0.96 (0.92–0.98)	1.45 (3.28)	4.01 (9.09)
	Mean 2 trials	66.52 (7.19) (48.41–85.54)	66.58 (7.08) (47.62–80.74)	0.96 (0.92–0.98)	1.48 (3.90)	4.10 (10.82)
	1 trial	66.51 (7.17) (48.42–85.49)	66.54 (7.09) (47.69–80.63)	0.92 (0.86–0.96)	1.98 (5.23)	5.48 (14.51)
PABD-tape	Mean 3 trials	4.08 (0.79) (2.47–7.05)	4.14 (0.83) (2.50–7.23)	0.95 (0.90–0.97)	0.18 (3.84)	0.51 (10.64)
	Mean 2 trials	4.08 (0.79) (2.47–7.05)	4.14 (0.83) (2.50–7.23)	0.95 (0.90–0.97)	0.18 (3.84)	0.51 (10.66)
	1 trial	4.06 (0.78) (2.50–7.00)	4.12 (0.82) (2.50–7.20)	0.90 (0.82–0.95)	0.25 (8.00)	0.70 (22.20)

^†^M, mean; SD, standard deviation; 95% CI, 95 percent confidence interval; SEM = SD*√ (1-ICC); MDC_95_ = SEM*1.96*√(2); MDC% = (MDC/Range of IMD values for session 1) * 100. Means, standard deviations, SEM, and MDC are reported in millimeters (caliper) or centimeters (tape)

The SEM, MDC, SEM%, and MDC% were calculated to determine measurement precision. SEM values ranged from 1.35 to 1.98, with the smallest RABD-caliper (2/3 trials) and the highest PABD-caliper (1 trial). MDC values ranged from 3.7 (RABD-caliper) to 5.48 (PABD-caliper). According to the MDC%, RA-caliper (Means 2/3 trials) and PABD-caliper (Mean 3 trials) had excellent precision, the others had acceptable [36]. Furthermore, based on the clinical acceptability of a SEM% value of less than 10% [38], all measurements were found to have acceptable precision.

### Construct validity

All IMD and MHQ data were normally distributed. Strong significant and moderate nonsignificant positive associations were found between the MHQ activities of daily living subscale and RABD-caliper (*r* = 0.38, *p* < 0.05) and the PABD-caliper (*r* = 0.27, *p* = 0.09) methods, respectively. Strong significant and poor nonsignificant positive associations were found between the MHQ activities of daily living subscale and RABD-tape (*r* = 0.40, *p* < 0.01) and the PABD-tape (*r* = 0.04, *p* = 0.80) methods, respectively.

## Discussion

This study investigated the reliability, precision, and construct validity of caliper and tape-measure IMD methods for assessing PABD and RABD in individuals with CMC1 OA and examined the impact of trial averaging on measurement consistency. Both caliper and tape-measure methods demonstrated excellent reliability and acceptable precision across all measurement conditions. Rheumatology professionals (e.g., physicians, nurse practitioners, and occupational therapists) can be confident that these methods are reproducible and precise in the context of CMC1 OA when administered across time by the same evaluator.

The assessment of the goniometric findings was not included in our study due to its previously established low reliability compared to distance-based measurements [14] and the concerns reported by various researchers about its use in measuring thumb abduction [13, 14, 16]. Further, we did not concurrently assess the reliability of the Pollexograph as an angular measurement method [13] because previous authors have noted its lower reliability relative to the IMD method [16]. Our study also excluded the inter-interphalangeal distance method, a distance-based method like the IMD method, because it may not be a true or repeatable measure of motion at CMC1 due to radial-drift of the proximal phalanx and metacarpophalangeal hyperextension, which are frequently seen in CMC1 OA patients [39].

Our study did not focus on the reliability of the IMD-caliper method for assessing RABD given that it has already been established to have excellent reliability in persons with healthy hands and those affected by CMC1 OA [13, 18]. In the study of healthy participants, the test–retest reliability of the IMD tape-measure method assessment of RABD for different raters ranged between 0.82 and 0.91 [14]. However, at the time of our study, the test–retest reliability of the IMD tape-measure method had not been investigated in persons with CMC1 OA. Similar to the results described by Holzbauer et al. [13] (ICC = 0.82–0.91), our findings support that this method yields excellent reliability. However, our slightly higher reliability (ICC = 0.93) may be attributable to the marking of anatomical landmarks which was not performed by Holzbauer et al. [14].

Previous studies investigating the test–retest reliability of the measurement of PABD using the IMD caliper method generally consisted of healthy individuals or persons with median or ulnar nerve injuries [13, 14]. Our study is the first to present the test–retest reliability of measuring the IMD in PABD using a caliper and tape measure in individuals with CMC1 OA. Our results were similar to the findings of other researchers [13, 14]. However, our results support that the reliability of the average of 2 and 3 trials is higher than those reported by others who studied other populations and used single trials [13, 14]. The reliability of the average of 2 or 3 trials using a tape-measure (ICC = 0.95), a tool accessible in every rheumatology clinic, was close to that of the caliper (ICC = 0.96) despite its lower resolution (1 mm) than that of the caliper (tenth of a mm). This demonstrated that the tape measure can be used for both RABD and PABD in assessing thumb abduction with excellent reliability. In addition, we think that our new assessment approach, which aims to minimize the effect of deformities on the assessment of PABD with tape measure in individuals with CMC1 OA, will contribute to both future research and clinical use.

According to our results, the reliability of the averages of 2 and 3 trials trended towards being higher than that of 1 trial however the reliabilities of the average of 2 trials appeared to be similar to that of 3 trials. Therefore, conducting at least two trials and using the average of these may lead to more reliable and precise results. It was also observed that more than one trial yielded better precision. Similar to what was previously found, the precision of the mean of 2 or 3 trials for IMD-caliper measurements of PABD was better than that of one trial, as was the precision of IMD-caliper measurements of RABD (RABD MDC%: 13.0, 9.1, 9.1) [19].

This study is the first to report the precision of tape measure measurements of thumb PABD and RABD in individuals with CMC1 OA. For PABD measurement (tape measure), the precision of the mean of 2 and 3 measurements was close to that of the caliper, but the precision of the 1 trial was notably lower than the mean of 2/3 measurements. The one-trial precision of the PABD was half that of the 2 and 3 trials, and for the RABD, the difference was about 50%. This indicated that the precision of the 1 trial was considerably lower. Therefore, as recommended in other hand function assessments (grip strength, pinch strength, etc.), performing the thumb abduction measurement with more than one trial may provide more precise and reliable results [14, 40–42]. This can be said for all measurements of thumb PABD and RABD with calipers or tape measures, but it is even more prominent for tape measures.

Finally, strong associations were observed between RABD measurements and disability scores. While no multivariate analyses were performed, the data would suggest that these measurements accounted for 20% (*r*^2^ = 0.20) of the variance in disability scores. These findings highlight the validity of using these measurements in the context of CMC1 OA.

## Limitations

Given that the methods employed in this study are standardized for individuals with CMC1 OA, their applicability to other patient populations is limited. Due to potential variations in precision across different brands and models of digital calipers and tape measures, further research using diverse equipment brands is necessary. The homogeneity of the study sample concerning race and ethnicity limits the generalizability of the findings.

## Conclusion

Both caliper and tape measure methods yield excellent reliability and acceptable precision. While 1 trial of any of the studied IMD measurement yields excellent reliability and acceptable precision, there is notable more measurement error. Therefore, we recommend that clinicians take the average of 2–3 measurements of any of the IMD approaches rather than 1 single measurement. We do not recommend caliper over tape measurements but also do not recommend they be used interchangeably. Finally, given the higher associations between RABD measurements and self-reported disability, RABD measurements may provide more clinically meaningful information but this decision should also take into consideration the client’s reported functional limitations and daily demands.

## Data Availability

The data that support the findings of this study are available from the corresponding author upon reasonable request.

## Declarations

### Artificial intelligence

Artificial intelligence was not used in any manner during the drafting of this manuscript.
